# The psychological problems and related influential factors of left-behind adolescents (LBA) in Hunan, China: a cross sectional study

**DOI:** 10.1186/s12939-017-0639-2

**Published:** 2017-09-02

**Authors:** Ye Man, Lv Mengmeng, Li Lezhi, Mao Ting, Zhang Jingping

**Affiliations:** 1 Department of Nursing, The Second Xiangya Hospital of Central South University, Changsha, Hunan People’s Republic of China; 2 Nursing School of Central South University, Changsha, Hunan People’s Republic of China

**Keywords:** Adolescent psychiatry, Life events, Coping, Social support, Family functioning, Self-esteem, Personality

## Abstract

**Background:**

Due to lack of companionship of parents, compared with non left behind children, left behind children (LBC) suffer from more psychological problems compared with children live with their parents. The aim of this study was to explore the mental health status and the relationship among psychological problems and the related factors of LBC.

**Method:**

Adopting delaminating-random-group sampling and using region, county, village (town) as sampling framework, we utilized Demographic Data Recording Form, Adolescent Self-Rating Life Events Check List, Scale of APGAR, Perceived Social Support Scale, Simplified Coping Style Questionnaire, Eysenck Personality Questionnaire, Self-Esteem Scale and Scale of Mental Health for Chinese Middle-school Student to assess 1309 left behind child in junior middle school students’ mental health in Hunan. Statistic description, Structural equation model was adopted to analyze the data.

**Result:**

There was a significant difference in score of psychological problems between LBC and non-LBC(F = 18.224, *P*<0.000). Life event was the major factor(*r* = .487) that affected psychological problems (path coefficient, PC = 0.08) directly and affect psychological problems indirectly through affecting passive coping (PC = 0.01)and family functioning(PC = 0.02); family functioning impacted psychological problems indirectly through affecting social support (PC = 4.89) and self-esteem (PC = 0.10); social support (PC = −0.02), passive coping (PC =0.07) and active coping PC = −0.04) affected psychological problems directly. Psychoticism (P) (PC = 0.11), Neuroticism (N) influenced psychological problems of LBC both directly (PC = 0.04) and indirectly through affecting self-esteem (PC: P:-1.87; N: -0.83), while Extraversion/Introversion (E) (PC = 0.21) only impact psychological problems indirectly through self-esteem. Altogether, these variables accounted for 50.2% of total variance of psychological problems (F = 130.470, *P* = 0.000) for LBC.

**Conclusion:**

In this research we proved that LBC have more sever psychological problems than non-LBC. We also identified the direct and indirect influential factors of psychological problems of LBC. The findings had important implications for prevention policies and interventions to promote mental health of LBC.

## Background

In China, it was estimated that 269 million rural residents have moved to cities by 2013 according to the China Women’s Federation in search of better job opportunities or wage [1]. However, most of them still are unable to keep their children with them in the cities and thus have to leave them behind with one single parent (usually the mother), grandparents, other relatives or friends in rural areas. LBC are defined as children who are under 18 years old and are left at home with both or one of their parents migrate to urban areas for at least 6 months. According to the National Women’s Federation population data projections, in China there are more than 61 million LBC in rural areas, which represents 37.7% of the total rural children in China in 2013 [1]. The increasing number of LBC has captured the attention of society [2] .

LBC live in incomplete families or unstable family environment, have inadequate parental supervision, and get less love and caring from parents, all of which make LBC much more easier to be neglected [3]. Worse school performance [4], lower nutrition [5, 6], worse physical health or physical well-being [7], higher risk of injury [8, 9]and higher proportion of poor behavior [10–12], such as drinking alcohol, smoking and internet addiction have been documented on LBC compared with non-LBC in China. Recent studies [12–14] found that mental health is damaged even more than physical health in the LBC.

It [15] reported that the LBC were 2.5 times (95% CI: 1.7, 3.5) more likely to suffer from loneliness and 6.4 times (95% CI 4.2, 9.7) more likely to be very lonely compared with non-LBC. They also had a higher likelihood of depression risk than controls (migrant fathers: OR = 3.42; migrant mothers: OR = 2.62; migrant parents: OR = 2.73) [16]. LBC were less socially adjusted and had more feeling of abandonment or neglect [17], therefore they had more psychopathology (especially hyperactivity) and less pro-social behaviors than the controls [18]. A huge attention is urgently needed among school and social care authorities regarding psychological problems in LBC and relevant risky factors in order to take preventive measures.

Previous studies have found a possible connection between psychological problem and socio-demographic indicators in left-behind status. LBC, who were left behind early in life, for longer periods, hadn’t an elder sibling, were in the care of young caregivers or non-relatives with poor education and low socioeconomic status, had more psychological problems [10, 18]. However, these factors are related to their family structures and environments, which cannot be easily intervened by school and society. A few studies found that LBC with less negative life events [19], more social support [20], better family function [18, 21] had less psychological problems, however, these factors were studied separately in association with mental health. Personality [22], coping style [19], self-esteem [23] also influence individuals’ mental health but still not studied in LBC.

Literature review demonstrated that social support not only impact the psychological status directly, but also play a buffer role in psychological status and negative life events, after suffer from negative life events adolescents who get more social support experience fewer negative emotional [24]. Coping style work as a mediator in the relationship between stress life events and psychological problems, and the path coefficient of positive coping between life events and negative emotion both are negative [25], meanwhile personality may work as a buffer in the impact of childhood traumatic life events on outcome in patients with mental disorders [26]. With self-esteem as influencing factors of mental health problems of LBC, the occurrence of life events decrease the level of self-esteem, self-esteem may work as a mediator between life events and mental health status of LBC [27]. And it reported that family function and life events are correlate with each other [28], coping style is one of the influential factors family function [29]. Via literature review, it indicated that self-esteem is an important psychological resource contains ability and value, is the core of individual mental health, which can be influenced by personality [30], family function [31], coping style [32], social support [33], then may effect the mental health status of individual. Therefore, the relationship among these variables maybe multidimensional and complex, which cannot be explained only by multiple linear regression, while few of the present studies have investigated the latent relationships among these variables.

According to J.C. Coyne’s theory of psychological stress. Stress includes stressors, mediator variables and physiological and psychological reactions [34]. The main stressors include life events, sudden traumatic chronic tension. Mediator variables are cognitive evaluation, coping style, social support and sense of control. The psychological and physiological responses are mainly the changes of various emotional reactions and changes in physiological and biochemical indicators, the most common emotional reactions are depression and anxiety. According to the literature review on the mental health status and the influencing factors of the LBC, the stressors of the left-behind children include the various negative life events and the general condition of the LBC (parents going out, guardianship, school situation), stress mediators include family factors (family care), social factors (social support), personal factors (coping style, personality, self-esteem).

**Fig. 1:**
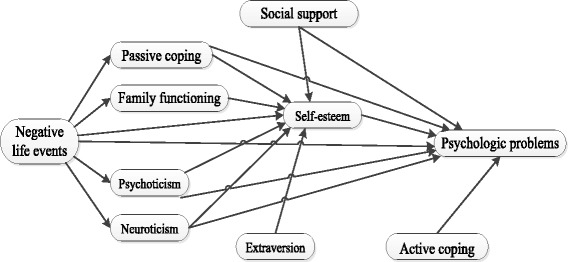
The assumed model of relationship among psychological problems and related influential factors in LBA

On the basis of literature review and the theory of psychological stress, we got the hypotheses: (1) Life events as the main stressor not only impact the mental health status of LBC directly, but also effect the LBC via some subjective and objective factors such as social support, self-esteem, family function, coping style, personality and so on. (2) Social support, parenting style, coping style, family care, personality have a direct effect on the mental health of LBC, and can also impact the psychologic status of LBC via self-respect. The initial psychological stress model of LBC was established based on the hypotheses (Fig. 1). This study used initial psychological stress model to investigate the mental health status and influencing factors of LBC, and designed the research and investigation.

## Methods

### Procedure and subjects

A stratified cluster cross-sectional survey was conducted in five rural counties of Hunan province, each from Central Hunan, East Hunan, South Hunan, West Hunan and North Hunan respectively. Then, three junior middle schools from each county were selected accordingly. With permission of principals and teachers from these 15 schools, an informed consent form was given to those students aged 12 to 18, who did not have cognitive impairments, were not orphans or from single-parent families. Ethical committees from Central South University, the Health Department of Hunan provinces and all five counties approved the research protocol.

The present study aims to build the structural equation model of the LBC’s/non-LBC’s mental health status and related influencing factors. According to the requirement of structural equation model, the baseline of the sample is 200, we should increase at least 5 to 10 people when we increase one variable. We had 54 variables in our study, so we need at least 740 people. Considering the stability of the model, the sample size should be more than 900. Taking into account the balance of the two sets of sample size, non-left behind children should be also more than 900. So the simple size of all the sample should be 1800 at least.

A total of 3000 adolescents agreed to participate in the survey and completed it in their classrooms without the presence of teachers and with the administration of a trained research assistant. 294 withdrew and 300 omitted answering 20% or more of the questions; 2406 adolescents completed the study, resulting in an effective response rate of 80.0%. Of 2406 students, 1309 students who responded “No” to the question “Has your father/mother NOT been living with you for at least six months in a row?” and gave “working” as the reason of separation were identified as LBC. The remaining 1097 students whose parents lived with them full time (45.6%) were non-LBC. There are three types of LBC: 458 students (19.0%) with fathers who left to seek employment were cared for by their mothers; 118 students (4.9%) with mothers who left home were cared for by their fathers; and 733 (30.5%) students with both parents who left to find work and they lived with grandparents or other relatives.

### Measures

The demographic data collection was obtained with a self-designed questionnaire. Characteristics of the sample included gender, age, grade, parents’ educational level, parents’ occupation and relationship with their caregivers.

The Scale of Mental Health for Chinese Middle-school Student (SMHCMS) [32] was used to evaluate young people’s psychological problems in China. This scale includes 60 items, which are set on a 5-point Likert scale from 0 (None) to 4 (very severe). It includes ten 6-item subscales: Obsessive-compulsive, paranoia, hostility, tension and interpersonal sensitivity, depression, anxiety, study pressure, maladjustment, emotional imbalance, and psychological imbalance. This scale has shown good reliability and validity in a survey on more than 20,000 middle school students in China [35] with relevant coefficient of all subsca les and total scale between 0.77 and 0.87.

The adolescent Self-Rating Negative life events Check List (ASLEC) presents 27 common, negative life-events in adolescent’s life. The scale aims to assess whether the negative events occurred on the participant as well as the effects, if any, of the stressful negative life events in the past year. Responses are made based on a range, from 0 (not at all) to 4 (very much). The scale reportedly has good reliability and validity with Cronbach α 0.85 and Spearman-Brown split-half reliability coefficient 0.88 [36].

Social support from family, friends and significant others was measured using the 12-item Perceived Social Support Scale (PSSS) [37], with a higher score indicating higher social support. Responses range from 1 (strongly disagree) to 7 (strongly agree). It has shown good reliability and validity in various samples [38] and had been used in China [39].

The APGAR developed by Smilkstein [40] was used to assess the perception of family functioning through examining his/her satisfaction degree towards family relationships with five items on a 3-point scale ranging from 0 (hardly ever) to 2 (almost always). “APGAR” is comprised of the first letter of five parameters of family functioning: adaptability, partnership, growth, affection, and resolve. Higher scores indicate better family functioning. The family function is good when the total score is between 7 to 10, moderately obstacle when the total score is between 4 and 6, and seriously obstacle when between 0 and 3. It has a well-established validity and reliability, with the Cronbach α at 0.84 and 2-week test–retest reliability at 0.70.

Coping style was measured by the 20-item Simplified Coping Style Questionnaire (CSQ) [41]. Responses for each question range from 0 to 3 (0 = never do, 1 = seldom do, 2 = often do, 3 = always do). This scale includes two sub-scales to assess active coping (item 1 to 12) and passive coping (item 13 to 20). The higher score of each dimension indicated frequent usage of this type of coping. The internal consistency measured by Cronbach α was found to be 0.90, 0.89 and 0.78 for subscale and the whole scale respectively.

The Eysenck Personality Questionnaire (EPQ) [42] includes 88 items, which consists of four sub-scales: psychoticism (P), extraversion/introversion (E), neuroticism (stability/emotionality, N), and lying (the revelation of falsehoods, L). The scale has good psychometric properties with the retest reliability values for each subscale being 0.67, 0.88, 0.80, and 0.78, respectively and has been applied to elementary and middle school students in China.

The ten-item Rosenberg Self-Esteem Scale (RSES) [43] was used to measure self-esteem or overall feeling of self-worth or self-evaluation. Responses range on a 4-point scale from 1 (strongly disagree) to 4 (strongly agree). Overall self-esteem factor can be calculated with the sum score ranging from 10 to 40. A higher score indicates higher self-esteem. Internal consistency reliabilities, based on Cronbach α, ranged from .77 to .88 for various populations and the test–retest correlation over 2 weeks was .85 [44].

### Data analysis

Standard descriptive analyses were performed in the first step. All the scales used in this study were also checked for their distributional properties and Cronbach α, normal distributions and good internal consistency reliabilities were found. The mental health status of students was compared between LBC and non-LBC using covariance analysis, because there were statistical difference between the demographic data of the two groups’ students. Next, multiple linear regression was used to analyze the direct impact of negative life events, self-esteem, social support, coping style, family APGAR on psychological problems. Then, we explored the associations among these influential factors of psychological problems using Pearson’s correlation analysis, univariate regression and bivariate regression. The above analyses were performed by the means of SPSS for windows 18.0 software package. After that, we built the assumed relationship model. Finally, structural equation modeling was performed by using Linear 8.7 to build the final relationship model of the related major factors of mental health in LBA. All tests were 2-tailed; *P* < 0.05 was considered significant.

## Results

### Descriptive statistics

Of 1309 LBC students, 315(24.1%) were from North Hunan, 270 (20.6%) from South Hunan, 295 (22.5%) from Central Hunan, 295(22.5%) from West Hunan, and 134 (10.2%) from East Hunan. There were 661 boys(50.5%)and 648(49.5%) girls. The age range was 12 to 17 years, with an average of 14.44 ± 1.14 years. There were 405(30.9%) adolescents in grade 7, 419(32.0%) in grade 8, and 485(37.1%) in grade 9. ANOVA analysis (in Table 1) showed that there was a significant difference among LBC with different age (*P* = 0.004), grade (*P* = 0.000) and father’s education ((*P* = 0.049) in score of psychological problems. There was no significant difference among LBC with different gender, parents out for work, mother’s education, father or mother’s career and relationship with caregivers in score of psychological problems (*P* > 0.05). Then the covariance analysis revealed that there was a significant difference (F = 18.244, *P* = 0.000) in score of psychological problems between LBC and non-LBC (Table 2). Tables 3 and 4 showed the descriptive statistics for the related influential variables and psychological problems (including ten subscales) of LBC.

**Table 1:** Comparison of psychological problems of LBA with different Socio-economic indicators

Variables	Group	Number	%	Mean $\left(\overline{X}\right)$	SD *(S)*	F	P
Age	12	42	3.2	2.04	0.57	3.516	0.004
	13	224	17.1	2.06	0.54		
	14	373	28.5	2.22	0.59		
	15	413	31.6	2.21	0.55		
	16	240	18.3	2.20	0.52		
	17	17	1.3	2.25	0.51		
Gender	Male	661	50.5	2.16	0.54	0.911	0.340
	Female	648	49.5	2.20	0.57		
Grade	Grade 7	405	30.9	2.08	0.56	10.070	<0.001
	Grade 8	419	32.0	2.26	0.57		
	Grade 9	485	37.1	2.20	0.53		
Parents out for work	Only father out for work	458	35.0	2.17	0.55	0.157	0.855
	Only mother out for work	118	9.0	2.19	0.56		
	Both parents out for work	733	56.0	2.19	0.56		
Father’s education	1-6 year	297	22.7	2.23	0.55	2.621	0.049
	7-9 year	763	58.3	2.14	0.55		
	10-12 year	240	18.3	2.23	0.58		
	>12 year	9	.7	2.10	0.41		
Mother’s education	1-6 year	499	38.1	2.21	0.55	0.631	0.595
	7-9 year	681	52.0	2.16	0.55		
	10-12 year	123	9.4	2.17	0.60		
	>12 year	6	.5	2.31	0.63		
Father’s job	worker	481	36.7	2.16	0.57	0.733	0.569
	farmer	595	45.5	2.20	0.53		
	intelligentsia	13	1.0	2.35	0.55		
	government officer	17	1.3	2.21	0.77		
	others	203	15.5	2.18	0.60		
Mother’s job	worker	279	21.3	2.13	0.58	1.831	0.120
	farmer	796	60.8	2.20	0.54		
	intelligentsia	23	1.8	2.04	0.58		
	government officer	7	.5	1.88	0.65		
	others	204	15.6	2.22	0.60		
frequency of communication with parents	Never	34	2.6	2.22	0.55	0.171	0.953
	Once everyday	116	8.9	2.15	0.62		
	once within in week	367	28.0	2.18	0.53		
	once in a week to a month	171	13.1	2.17	0.53		
	Unset frequency	621	47.4	2.19	0.57		
Relation with caregivers	Father	85	6.5	2.14	0.58	0.415	0.913
	Mother	437	33.4	2.17	0.54		
	grandparents	565	43.2	2.18	0.56		
	grandparents in law	142	10.8	2.20	0.58		
	Uncles	27	2.1	2.20	0.56		
	Aunts	40	3.1	2.20	0.50		
	Brothers or sisters	7	.5	2.20	0.49		
	Themselves	6	.5	2.38	0.96		

Note: ANOVA analysis was used to compare the mean score of psychological problems among different groups of LBA

**Table 2:** Covariance analysis of the mental health status of LBC and Non-LBC

Variables		Mean $\left(\overline{\boldsymbol{X}}\right)$	SD	F	P
Obsessive-compulsive	non-LBC	2.126	0.569	10.629	0.001
	LBC	2.212	0.603		
Paranoia	non-LBC	2.001	0.683	8.416	0.004
	LBC	2.089	0.663		
Hostility	non-LBC	1.934	0.744	13.733	0.000
	LBC	2.051	0.784		
Tension and interpersonal sensitivity	non-LBC	2.098	0.692	8.667	0.003
	LBC	2.198	0.713		
Depression	non-LBC	2.015	0.694	15.146	0.000
	LBC	2.145	0.721		
Anxiety	non-LBC	2.108	0.745	19.824	0.000
	LBC	2.254	0.758		
Study pressure	non-LBC	2.185	0.794	17.707	0.000
	LBC	2.331	0.794		
Maladjustment	non-LBC	2.174	0.670	6.422	0.011
	LBC	2.243	0.673		
Emotional imbalance	non-LBC	2.201	0.690	16.018	0.000
	LBC	2.322	0.703		
Psychological imbalance	non-LBC	1.891	0.657	6.445	0.011
	LBC	1.969	0.689		
Mental health status	non-LBC	2.073	0.567	18.244	0.000
	LBC	2.181	0.558

**Table 3:** Descriptive statistic of the study variables

	Life events	Social support	Active coping	Passive coping	Family functioning	Psychoticism	Extroversion	Neuroticism	Lying	Self-esteem	Psychological problems
Mean	49.30	59.27	1.58	1.14	4.80	4.96	16.38	10.94	10.77	27.92	2.18
SD	13.44	11.78	.46	.49	2.32	3.26	3.88	4.83	3.76	3.99	.56
Range	0–108	12–84	0–3	0–3	0–10	0–18	0–25	0–23	0–22	10–40	0–4

**Table 4:** Descriptive statistic of subscale score of psychological problems

	Obsessive-compulsive	Paranoid	Hostilities	Interpersonal sensitivity	Depression	Anxiety	Study pressure	Adverse application	Emotional instability	Psychological Imbalanc
Mean	2.21	2.09	2.05	2.20	2.14	2.25	2.33	2.24	2.32	1.97
SD	0.60	0.66	0.78	0.71	0.72	0.76	0.79	0.67	0.70	0.69
Range	0–4	0–4	0–4	0–4	0–4	0–4	0–4	0–4	0–4	0–4

### Multiple linear regression analysis

With age, gender, father’s education, negative life events, family functioning, social support, passive coping, active coping, Psychoticism, Extraversion/Introversion, Neuroticism, Lying and self-esteem as “independent variables”, psychological problems as “dependent variable”, “stepwise” multiple linear regression analysis (Table 5) showed that all the variables except age, gender, father’s education and Lying enter the linear regression equation. “B” referred to the Regression Coefficient, represented the difference in the predicted value of the psychological problems for each one-unit difference in a influencing factor, if other factors remain constant; “Beta” referred to Standardized Regression Coefficient. “Beta” was the estimates resulting from a regression analysis that have been standardized so that the variances of dependent and independent variables were 1. The standardized regression coefficient (R^2^ = 0.502) shows that in the linear regression equation all the variables accounted for 50.2% of total variance of mental health (F = 130.470, *P* = 0.000) for LBC.

**Table 5:** Multiple linear regression analysis of the study variables on mental health of LBA

Dependent variable	Independent variables	B	Beta	t	P
Psychological problems	Negative Life events	0.130	0.316	15.042	0.000
	Social support	−0.003	−0.072	−3.265	0.001
	Active coping	−0.103	−0.085	−3.578	0.000
	Passive coping	0.211	0.185	8.524	0.000
	Family functioning	0.019	0.067	2.693	0.007
	Psychoticism	0.020	0.116	5.224	0.000
	Extraversion/Introversion	−0.008	−0.053	−2.364	0.018
	Neuroticism	0.037	0.320	13.864	0.000
	Self-esteem	−0.018	−0.117	−5.167	0.000

Note: Stepwise multiple linear regression analysis was used to predict the related influential factors of psychological problems

Assumed predictors: age, gender, father’s education, negative life events, family functioning, social support, passive coping, active coping, Psychoticism, Extraversion/Introversion, Neuroticism, Lying and self-esteem

“B” refers to the Regression Coefficient, represents the difference in the predicted value of the psychological problems for each one-unit difference in a influencing factor, if other factors remain constant; “Beta” refers to Standardized Regression Coefficient. “Beta” is the estimates resulting from a regression analysis that have been standardized so that the variances of dependent and independent variables are 1

### Mediating effect

Table 6 shows the correlation matrix for the variables. Negative life events, family functioning, passive coping, Psychoticism, Neuroticism, self-esteem and psychological problems were significantly related to each other. Then we conduct “Stepwise regression coefficient” to test the mediating effect. In Tables 7 and 8 we identified the mediating variables between influencing factors and psychological problems. With psychological problems as dependent variable, bivariate regression found that Beta of negative life events decreased but were still significant after adding family functioning, passive coping, Psychoticism, Neuroticism and self-esteem as independent variables (Table 7). All the variables of Family functioning, passive coping, Psychoticism, Neuroticism and self-esteem partly mediate the influence of negative life events on psychological problems of LBC, and negative life events still had a direct effect on psychological problems. Family functioning, social support, active coping, passive coping, Psychoticism, Neuroticism, Extraversion/Introversion, self-esteem and psychological problems were significantly related to each other. With psychological problems as dependent variable, bivariate regression found that Beta of social support, active coping, passive coping, Psychoticism, Neuroticism decreased but still significant while Beta of APGAR and Extraversion/Introversion became insignificant when adding self-esteem as an independent variable (Table 8). It means that self-esteem partly mediates the influence of social support, active coping, passive coping, Psychoticism, Neuroticism on mental health status of LBC while self-esteem completely mediated the influence of APGAR and Extraversion/Introversion on psychological problems of LBC.

**Table 6:** Correlation matrix of the study variables

	Life events	Social support	Active coping	Passive coping	Family functioning	Psychoticism	Extroversion	Neuroticism	Self-esteem	Psychological problems
Life events	(0.854)	−.038	.017	.188***	−.100***	.131***	.016	.310***	−.112***	487***
Social support		(.852)	.352***	.023	.369***	−.137***	.251***	−.156***	.246***	−.149***
Active coping			(.730)	.273***	.368**	−.090**	.331***	−.079**	.294***	−.044
Passive coping				(.685)	.070*	.192***	.032	.235***	−.097***	.383***
Family functioning					(.728)	−.102***	.274***	−.183***	.259***	−.098***
Psychoticism						(.730)	−.132***	.301***	−.268***	.331***
Extroversion							(.696)	−.088**	.375***	−.113***
Neuroticism								(.809)	−.291***	.548***
Self-esteem									(.705)	−.316***
Psychological problems										(.854)

Note: Pearson Correlation was used to analyze the Correlation among the study variables

*Correlation is significant at the 0.05 level (two-tailed)

**Correlation is significant at the 0.01 level (two-tailed)

***Correlation is significant at the 0.001 level (two-tailed)

() Cronbach Alpha of the scale or subscale in present study

**Table 7:** Comparison of Beta of negative life events on psychological problems in LBA after adding mediating variables

Mediating variables	Casual variables	Beta1	Beta2	t	P
Family functioning	Life events	0.487	0.482	19.831	0.000
Passive coping	Life events	0.487	0.430	18.538	0.000
Psychoticism	Life events	0.487	0.452	19.466	0.000
Neuroticism	Life events	0.487	0.440	19.661	0.000
Self-esteem	Life events	0.487	0.454	19.440	0.000

Note: Beta1: Beta of negative life events in the univariate regression equation with psychological problems as dependent variable

Beta2: Beta of negative life events in the bivariate regression equation with psychological problems as dependent variable after adding mediating variables

**Table 8:** Comparison of Beta of Casual variables on psychological problems in LBA after adding self-esteem

Mediating variables	Casual variables	Beta1	Beta2	t	P
Self-esteem	Family functioning	−0.098	−0.025	−0.916	0.360
Self-esteem	Social support	−0.150	−0.080	−2.946	0.003
Self-esteem	Passive coping	0.382	0.350	14.225	0.000
Self-esteem	Psychoticism	0.331	0.279	10.866	0.000
Self-esteem	Extraversion	−0.112	−0.004	−0.127	0.899
Self-esteem	Neuroticism	0.548	0.499	20.706	0.000

Note: Beta1: Beta of casual variable in the univariate regression equation with psychological problems as dependent variable

Beta2: Beta of Casual variable in the bivariate regression equation with psychological problems as dependent variable after adding self-esteem

### Structural equation model

**Fig. 2:**
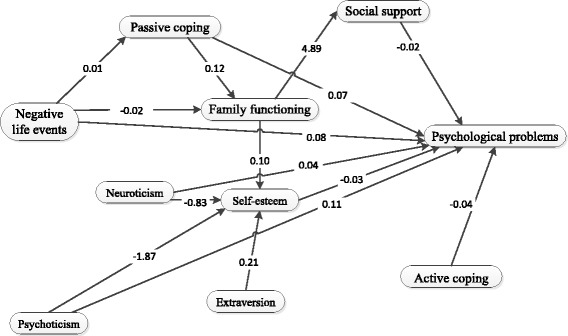
The simplified model of relationship among psychological problems and related influential factors in LBA

On the basic of the initial psychological stress model (Fig. 1), maximum likelihood method was used to estimate parameters and built and modify the structural equation model using LISREL8.70. According to the revised index, after three amendments, in turn reduce the path between the life events to emotional stability, life events to mental quality, negative response to self-esteem, and increase the path between passive coping to family care, family care to social support. Finally, we obtained the best model M4(X2/df < 3,CFI > 0.90,IFI > 0.90, GFI > 0.90,RMSEA < 0.05) after amending for three times. The simplified model M4 was shown as Fig. 2. The simplified model (Fig. 2) show the relationships and path confidence (PC) among negative life events, family functioning, social support, passive coping, active coping, Psychoticism, Neuroticism, self-esteem and psychological problems in LBC. Model fit indexes are listed in Table 9.

**Table 9:** The fit indicators of different models

Model	X2/df	CFI	IFI	GFI	RMSEA
M1	3.58	0.91	0.91	0.87	0.050
M2	3.47	0.92	0.92	0.88	0.047
M3	2.97	0.92	0.92	0.88	0.046
M4	2.74	0.92	0.92	0.90	0.046

Note: Compared to model 1, direct paths from negative life events to Neuroticism and Psychoticism were deleted in Model 2; direct paths from passive coping to self-esteem was deleted in Model 3; direct paths from passive coping to family functioning and direct paths from family functioning to social support was added in Model 4

## Discussion

Our study confirmed the evidence that psychological problems were serious among adolescent LBC with average score being 2.1816 ± .55811, which means that they had mild psychological problems as a whole, meanwhile, compared with non-LBC, the LBC have more severe psychological problems. As a result of being in a crucial stage of psychological development and without parents living with them for longer time, LBC who are older than 14 and in grade 8 had more psychological problems. Other studies found that those LBC who were brought up by grandparents, and the low frequency of communication with parents was prone to encounter more severe psychological problems than other left-behind [10, 18]. However, our results demonstrated a different picture. It may be because that most of those in our study were brought up by the grandparents (43.2%) or mothers (33.4%), or with both parents absent (56.0%). And most of them communicated with their parents on unset frequency (47.4%) or less than once weekly (15.7%). Moreover, some factors such as the duration of parents being away from home and the methods and contents of communication may also contribute to the impact of mental health on LBC. Furthermore, other factors such as negative life events, family functioning, social support, coping style and self-esteem can influence their mental health greatly, and accounted for 50.2% of total variance of their psychological problems.

Negative life events were the major factors that predict the LBC’s psychological problems (Beta = 0.316, PC =0.08). LBC were in the contradiction of rapid physical development and lagging behind psychological development and experienced more negative life events from the absence of their parents. Research [45] has indicated that grandparents or other relatives may spoil the children or display violent behavior toward the children in their care and the “foster” child may feel less access to the guardian’s concern. Even those cared for by a single mother or father, can neither get enough care and supervision nor communication with each other often [46] because of not having enough time and energy to take care of them. LBC are more sensitive to the views and perspectives of people in their environment [46], but they cannot learn from their parents how to handle the interpersonal relationships successfully as their parents are not living with them and communicate with them infrequently [47]. Studies also found that they had more risks of injury [9]. Moreover, the absentee parents felt compelled to work in cities to provide better financial support for their children’s education and living conditions and hoped that their children would get a better job in the future through better education. They have more anxiety about their children’s school achievement, which imposes more study pressure on LBC. In this study, we found that the LBC had the most problems on study pressure, followed by emotional instability, anxiety and obsessive-compulsive.

We also found from the structural equal model that negative life events can not only directly affect the psychological problems (PC = 0.08), but also can indirectly affect psychological problems by influencing passive coping (PC = 0.01) and family functioning (PC = − 0.02). Face more negative life events but without the ability to get much help and guidance, the LBC tend to take a negative outlook to the conditions surrounding them, including the parents’ absence. Therefore, they will perceive worse family functioning, which also lead to more psychological problems. In this study, passive coping was positively related to while active coping negatively related to psychological problems. Adolescents who adopt a proactive approach to dealing with difficulties would talk to others, telling why they are dissatisfied and asking their relatives or friends for help. All of those factors would help them build a relationship network, which would be helpful for them to access social support. They can get more help to go through difficult times. While LBC with passive coping cannot make use of the family support, they will take a negative outlook at their parents’ absence, thus influence their perceived family functioning and social support.

Stable family circumstance and functioning can help to promote psychological development of adolescents [48]. In our study, family functioning was negatively correlated with psychological problems of LBC. However, it only influenced psychological problems indirectly through affecting social support (PC = 4.89) and self-esteem (PC = 0.10). Families with high APGAR can support democratic education to the children, also tend to be strict with their children; as a result of respecting children’s personality, these families also can understand their interests and requirements as well as can provide superior material conditions to them, and thus, can reduce negative psychological reaction, all of which were helpful to the development of mental health. Previous studies [31] found that family functioning was significantly related to self-esteem and was a strong predictor of self-esteem in adolescents. In this study we also found that family functioning can also affect LBC’s psychological problems through influencing their self-esteem. LBC who perceived better family functioning had higher self-esteem, and therefore better mental health.

Although the parents may not live with them, family functioning was still the most important factor that is significantly related to the social support (*r* = 0.369). LBC were more eager to get concern and support from their parents, which can help them get more information about the daily living, expand their knowledge, develop the ability to analyze and solve problems, establish good relationship with partners; at the same time it can give them more positive feelings, increase self-confidence, and encourage them to participate actively in social activities and work even harder. In our study, social support was negatively correlated with psychological problems (*r* = − 0.151), as it can intermediate the influence of family functioning on psychological problems and affect psychological problems directly (PC = −0.02). The higher social support scores, the healthier the LBC’s psychological problems. This findings were similar to the findings of former studies [49].

Psychoticism (*r* = 0.331; PC = 0.11) and Neuroticism (*r* = 0.548; PC = 0.04) were the major personality factors that affect psychological problems. They not only affected psychological problems directly, but also affected their psychological problems indirectly through intermediary role of self-esteem (PC: P:-1.87; N: -0.83). With Psychoticism and Neuroticism increasing, self-esteem decreased, the psychological problem scores increased. The higher score of psychoticism and neuroticism have worse mental health. However, Extraversion/Introversion did not affect their psychological problems directly, but affected their psychological problems through their self-esteem (PC = 0.21). LBC who tend to be introverted had lower self-esteem and worse mental health.

Self-esteem played a more important intermediate role than personality in the development of LBC mental health development. Self-esteem is often used to describe the perception of the individual’s self-worth. It is intended to reflect the individual’s basic views of themselves, the world and the future. It is an important component of the individual self-system, and it is vital in restoring or maintaining both mental and physical health [23]. In this study, self-esteem could affect the psychological problems directly and it can intermediate the impact of personality and family functioning on psychological problems. Adolescents with higher self-esteem were more self-confident and more comfortable with teamwork and family functioning, which could help them avoid and defend the threat, such as failure or social exclusion.

Our study has several limitations. Firstly, this study cannot interpret cause-effect relationship and long-term effects such as any cross-sectional study. We attempted to find the direct and indirect major factors of psychological problems in LBC, using different statistics analysis methods including structural equation modeling. Secondly, all of the demographic and subjective assessment data were self-reported by students, this, however, may lead itself to reporting bias. In order to minimize reporting bias, students were asked to complete the questionnaire in class in their teachers’ absence and anonymity of the study was stressed by investigators. All the adopted questionnaires have been shown to have acceptable validity and reliability. Moreover, this study was conducted only in 15 junior middle schools in five rural area of Southern China; it did not cover LBC who are not enrolled in schools, which may limit the possibility to generalize the results across China although we tried to lessen these biases through enlarging samples.

Despite these limitations, the findings in this study have some important implications for prevention policies and interventions to promote mental health among LBC in China and other developing countries with similar internal migration issues. In China according to our national condition and economic reality, it will be difficult to solve the problem of LBC in a short period of time [50], so we have a series of prevention policies about LBC. However, most of the policies focus on the education, living condition, financial support and physical health of LBC, few policies provide some instructions about improving the mental health status of LBC. At the same time due to the lack of professional policy and training, most of the social workers can not provide LBC with effective protections, especially for their mental health [50]. So our research can provide theoretical basis for policy-making in preventing the mental health status of LBC. Firstly, the Chinese government can set up professional social work organizations and volunteer organizations for LBC, collect the demographic data of the LBC and investigate the mental health status of the LBC, provide consultation for the children who have sever psychological problem; local governments should also take the initiative to their mental health, by way of setting up school psychological consultation rooms, provide free psychological consultation for LBC regularly, tell them how to cope with negative life events actively, take use of social support effectively and take a correct view of their merits and drawbacks, which can improve the mental health status the LBC. Second, parents and caretakers should pay more attention to communicating with LBC to help them perceive positive family functioning. Third, many concrete actions can also be taken with supports from government, schools, enterprises and international communities, including free consultations at boarding schools, family reunions, and clubs. Many recreational activities can also be developed to help LBC build good relationship with their classmates. Finally, along with the support, providing adolescents with opportunities for autonomous self/environmental exploration (e.g, online assessment tools, extracurricular opportunities) is beneficial for increasing their self-esteem. Further intervention studies are needed to explore how to cope with LBC’s psychological problems effectively.

## Conclusions

Our study has confirmed that there are significant difference between LBC and non-LBC in psychological problems. Also, through the structural equation model analysis, we build a left-behind junior high school students’ psychological problem influential factors model, which has a good fitting index. Social support, family caring, positive and negative coping styles, self-esteem are mediator factors of psychological stress of left-behind children, and self-esteem is the most important and direct influencing factor of mental state. Self - esteem can buffer the impact of personality, family caring on psychological status, and negative coping can cushion the impact of life events on mental status, while social support can buffer the impact of family care on psychological status. Our study can provide instructions for further studies to found out effective interventions to improve the mental health of LBC and can also help the government formulate policies to help left-behind junior high school students.

## Abbreviations

APGAR: comprised of the first letter of five parameters of family functioning: adaptability, partnership, growth, affection, and resolve
CSQ: Coping Style Questionnaire
EPQ: The Eysenck Personality Questionnaire
LBC: left behind children
PC: path coefficient
PSSS: Perceived Social Support Scale
RSES: Rosenberg Self-Esteem Scale
SMHCMS: The Scale of Mental Health for Chinese Middle-school Student
